# Treatment of anterior corneal scarring, following DSAEK graft failure, with combined graft exchange and phototherapeutic keratectomy

**DOI:** 10.1186/s40662-017-0078-6

**Published:** 2017-05-04

**Authors:** George Kymionis, Konstantinos Oikonomakis, Myrsini Petrelli, Konstantinos Andreanos, Andreas Mouchtouris, Ilias Georgalas

**Affiliations:** 1st Department of Ophthalmology, University of Athens, General Hospital of Athens “G.Gennimatas”, 154 Mesogion Av, Athens, 115 27 Greece

**Keywords:** DSAEK failure, Anterior corneal scar, Graft exchange, Phototherapeutic keratectomy

## Abstract

**Background:**

To present a method, alternative to penetrating keratoplasty, for the restoration of impaired corneal clarity with anterior stromal scarring following long-standing corneal graft failure.

**Case presentation:**

A 48-year old female who had previously underwent Descemet stripping automated endothelial keratoplasty (DSAEK) for the treatment of pseudophakic bullous keratopathy, presented with long-standing corneal oedema and anterior corneal scarring. A significant improvement in corrected distance visual acuity was demonstrated, as corneal clarity was restored following graft exchange and phototherapeutic keratectomy (PTK).

**Conclusions:**

The combination of corneal graft exchange and phototherapeutic keratectomy may represent an effective therapeutic option for long-standing corneal oedema with concomitant anterior corneal scarring after failure of a DSAEK graft.

## Background

Descemet stripping automated endothelial keratoplasty (DSAEK) has become the modality of choice for the treatment of corneal oedema arising from corneal endothelial diseases including Fuchs endothelial dystrophy and pseudophakic bullous keratopathy [1].

In cases of DSAEK graft failure or rejection, corneal graft exchange could be attempted. Nevertheless, when corneal oedema is left to ensue, the subsequent chronic corneal decompensation may result in anterior corneal fibrosis. The aforementioned complication limits the final visual outcome of a new DSAEK procedure and, thus, may alter the surgeon’s plan in favour of penetrating keratoplasty (PK).

## Case presentation

A 48-year old female sought medical consultation due to decreased vision of the left eye. Her past medical history included no necessity for spectacle correction at a younger age and phacoemulsification surgery of the left eye 4 years ago. Following cataract surgery, no improvement of her visual acuity was noticed due to pseudophakic bullous keratopathy. Therefore, the patient underwent DSAEK procedure in the left eye six months post cataract extraction. Restoration of good visual acuity following DSAEK that lasted for the next year was noticed; upon the aforementioned period the patient reported gradual deterioration of vision. Gradual visual impairment was attributed to graft failure.

The ophthalmic examination of the left eye revealed uncorrected distance visual acuity HM (hand movement) that could not be improved with spectacles. Slit-lamp biomicroscopy of the left eye showed corneal oedema accompanied by anterior corneal fibrosis.

**Fig. 1:**
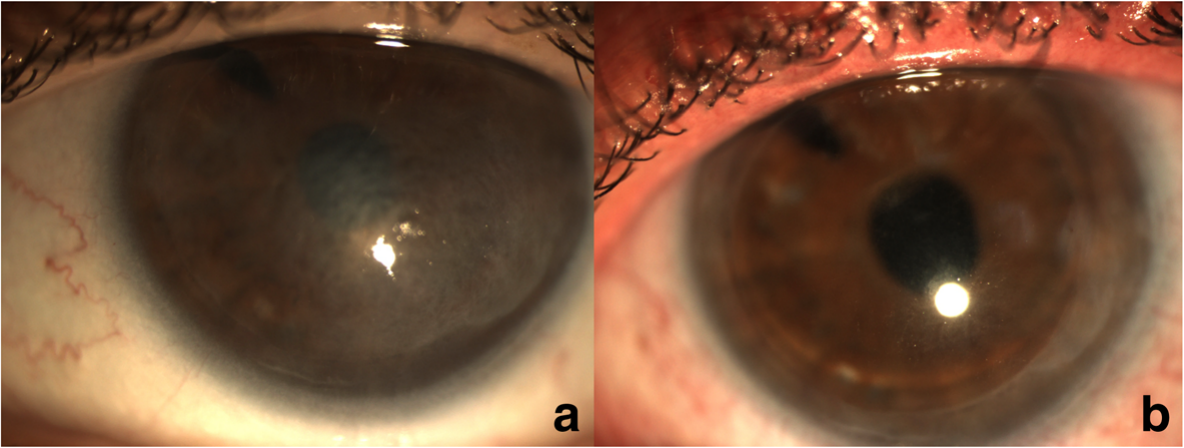
Post-redo-DSAEK slit lamp photography of the left eye prior to and following PTK. Slit lamp photography of the left eye. **a** Post-redo-DSAEK (1st month) slit lamp photograph demonstrating resolution of corneal oedema and the presence of anterior corneal scar. **b** Post-PTK slit lamp photograph (1st month) demonstrating clear cornea with absence of scar

**Fig. 2:**
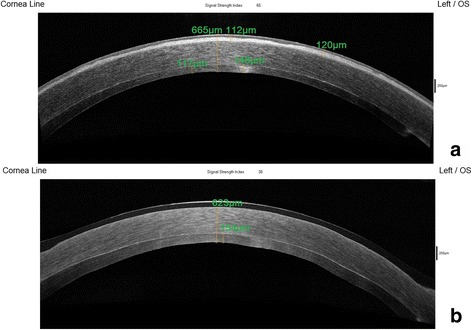
Post-redo DSAEK AS-OCT of the left eye prior to and following PTK. AS-OCT of the left eye at day of PTK treatment. **a** AS-OCT prior to laser ablation; corneal scar depth: 112 μm in the central cornea/120 μm in the mid-periphery, graft thickness: min 117 μm/max 146 μm. **b** Post PTK AS-OCT demonstrating resolution of scar

We proceeded with a second Descemet stripping endothelial keratoplasty (almost two years following failure of the primary graft) in order to replace the non-functional graft with a healthy one. The procedure resulted in complete resolution of corneal oedema within the first postoperative month (Fig 1a). Nevertheless, 6 months postoperatively, patient’s corrected distance visual acuity could not exceed 20/400 due to the ongoing presence of the corneal scar. Anterior segment optical coherence tomography (AS-OCT) was performed in order to establish the extension of fibrosis in the anterior stroma; in fact, the depth of the scar was estimated at 112 μm from the corneal surface centrally and at 120 μm in the mid-periphery (Fig 2a). A variety of other parameters were also calculated with the use of AS-OCT; central corneal thickness (involving both the scar and the graft) was measured 665 μm and graft thickness was measured 117 μm at its thinnest point (Fig 2a), Thus, the residual corneal tissue of the recipient was estimated at 548 μm. Our decision was to proceed with transepithelial phototherapeutic keratectomy (PTK) in a 7.0 mm-diameter treatment zone for a treatment depth of 120 μm. Treatment was initiated using the Wavelight EX500 femtosecond laser platform. Adjunctive mitomycin-C (MMC) 0.02% for 60 s was applied on the corneal surface. Prolonged antimetabolite application was performed in an effort to spare the patient from the increased risk of postoperative corneal haze that accompanies deep tissue ablations [2]. Postoperative AS-OCT was performed depicting the resolution of the scar (Fig 2b). Mixed eye drops containing antibiotic agent (chloramphenicol 0.5%) and cortisone (dexamethasone 0.1%) were administered 5 times daily for the first post-operative month, with gradual dose tapering over the following 6 months.

In the first postoperative month, anterior corneal fibrosis resolved (Fig 1b) and the patient’s corrected distance visual acuity reached 20/32, with a manifest refraction of −1.50 sph −2.00 cyl × 120°. The final myopic refractive outcome, as opposed to the expected hyperopia induced by DSAEK, was attributed to the myopic shift of the laser treatment. The aforementioned laser platform is scheduled by the manufacturer to induce myopia following PTK treatment (to compensate for the post-PTK hyperopic shift that was observed with previous treatment profiles). Central corneal thickness in the first postoperative month measured by ultrasound pachymetry was 517 μm.

## Discussion and conclusions

Corneal decompensation arising from a non-functional graft or from endothelial diseases such as Fuchs endothelial dystrophy [3] and pseudophakic bullous keratopathy, if severe or chronic enough, may lead to anterior corneal fibrosis. In the special case of long-standing graft failure, the preoperative finding of anterior fibrosis may lead the treating surgeon to proceed to PK instead of a new DSAEK procedure.

In our case, failure of DSAEK graft led to chronic corneal oedema with a central opacity that involved the anterior stroma. We decided against PK in an attempt to achieve not only satisfying post-keratoplasty visual acuity, as well as to obviate the increased morbidity of a full-thickness corneal graft. A two-step procedure that included DSAEK graft exchange and phototherapeutic keratectomy with adjunctive MMC was performed. To date, 6 months following PTK, no recurrence of the anterior corneal fibrosis has been observed.

Manual debridement of the fibrosis would be an alternative in case of a superficial scar. However, in the case of scar extension into the anterior stroma, manual peeling may give rise to deep corneal defects that may, in turn, lead to uneven healing and an unpredictable visual outcome [4]. In addition, the use of PTK ensures both the removal of the scar as well as a better refractive outcome due to homogeneity of the corneal surface.

In conclusion, DSAEK graft failure prompts consideration for timely restoration of subsequent corneal oedema, as chronic corneal decompensation can result in anterior corneal fibrosis. In the adverse event of graft failure with anterior corneal scarring, combined graft exchange and PTK should be considered in an effort to spare the patient from the increased morbidity of a PK graft and to achieve a greater visual outcome.

## Abbreviations

AS-OCT: Anterior segment optical coherence tomography
DSAEK: Descemet stripping automated endothelial keratoplasty
HM: Hand movement
MMC: Mitomycin-C
PK: Penetrating keratoplasty
PTK: Phototherapeutic keratectomy
